# Two for One: Concurrent Acquisition of Molluscum Contagiosum Infection and Scabies Infestation After a Single Sexual Encounter

**DOI:** 10.7759/cureus.20780

**Published:** 2021-12-28

**Authors:** Philip R Cohen

**Affiliations:** 1 Dermatology, University of California, Davis Medical Center, Sacramento, USA

**Keywords:** sarcoptes, surrepticius, sexually, scrotal, scabies, rejuvenation, penis, molluscum, mite, genital

## Abstract

Molluscum contagiosum and scabies are contagious conditions that can be acquired by incidental casual contact of a disease-free individual with an infected person. However, both the viral infection and the mite infestation can be transmitted sexually from the infected person to the unsuspecting recipient partner. A 20-year-old man without any infectious diseases acquired not only molluscum contagiosum but also scabies after a single sexual encounter with a female partner; hence, he developed two sexually transmitted diseases after one sexual episode: two for one. He presented for medical attention to evaluate the new white umbilicated papules on his penile shaft and red nodules on the corona and glans of his penis. A complete skin examination also revealed additional papules on his abdomen and suprapubic region and a burrow on his left index finger adjacent to the finger web. Diagnosis of the molluscum contagiosum infection was confirmed by observing molluscum bodies on the molluscum preparation taken from an abdominal papule and the scabies infestation was confirmed by noting not only mite eggs but also mite scybala (feces) on the scabies (mineral oil) preparation from the burrow. The molluscum contagiosum infection was treated with curettage of the lesions. The scabies infestation was treated with two treatments (each one week apart) of permethrin five percent cream overnight topical application from his neck to his toes. All his lesions resolved and did not recur. In conclusion, the development of a new sexually transmitted disease in an individual should prompt the clinician to evaluate the patient for additional sexually transmitted diseases since the patient may have acquired more than one infection from their partner during the sexual encounter.

## Introduction

Molluscum contagiosum is a deoxyribonucleic acid (DNA) poxvirus that appears as white to flesh-colored, dome-shaped, umbilicated papules. It occurs primarily in healthy children and adults; however, individuals with atopic dermatitis or immunosuppression--such as human immunodeficiency virus (HIV) infection--are at increased risk to acquire the infection. The viral infection is usually acquired by either incidental or sexual skin contact with an infected person; rarely, the virus can be acquired from contaminated fomites. Molluscum contagiosum lesions may subsequently be spread by autoinoculation [1-4].

Scabies is a parasitic infestation caused by the mite Sarcoptes scabieivar. hominis in humans. It can present with classic clinical manifestations or with an atypical presentation in patients with non-classic (surrepticius) variants. Scabies is transmitted by direct--either coincidental (such as assisting someone during their activities of daily living) or intentional (such as during a sexual encounter)--cutaneous contact usually for a minimum of five minutes with an infected person; rarely, fomites may be involved in the transmission of scabies from patients with the crusted variant [5-7].

A man with no prior history of sexually transmitted diseases is described who acquired not only molluscum contagiosum infection but also a scabies infestation after having a single sexual encounter with a woman. He sought medical attention for the lesions he identified on his penis; however, a complete skin examination discovered additional cutaneous manifestations of both conditions on other areas of his body. Therefore, individuals who present with a new sexually transmitted disease should be evaluated for the possibility of additional conditions that can be acquired sexually since the contagious person may have more than one disease.

## Case presentation

A 20-year-old man presented for evaluation of lesions on his penis. He had noted the new lesions to be present not only on the shaft, but also on the glans of his penis. The lesions were asymptomatic; however, he subsequently acknowledged that he had developed generalized itching that was worse in the evenings.

**Figure 1:**
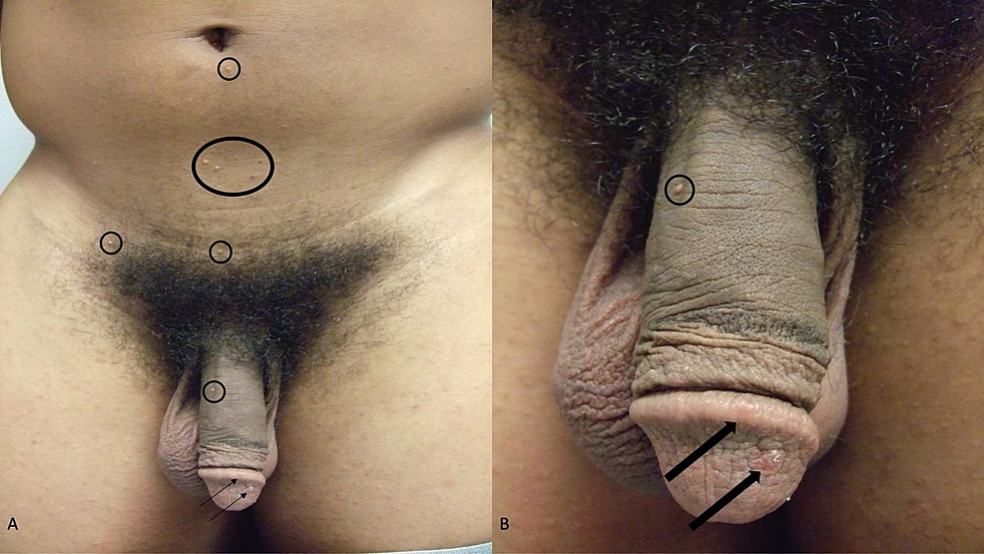
Lesions of molluscum contagiosum infection and scabies infestation on the abdomen, groin, and penis Distant (A) and closer (B) views of concurrently present molluscum contagiosum papules (within black ovals) and red scaling scabies nodules (black arrows) on the body of a 20-year-old man. The molluscum contagiosum lesions are on the abdomen below the umbilicus, the suprapubic region, and the penile shaft. The scabies lesions are on the corona and glans of the penis.

**Figure 2:**
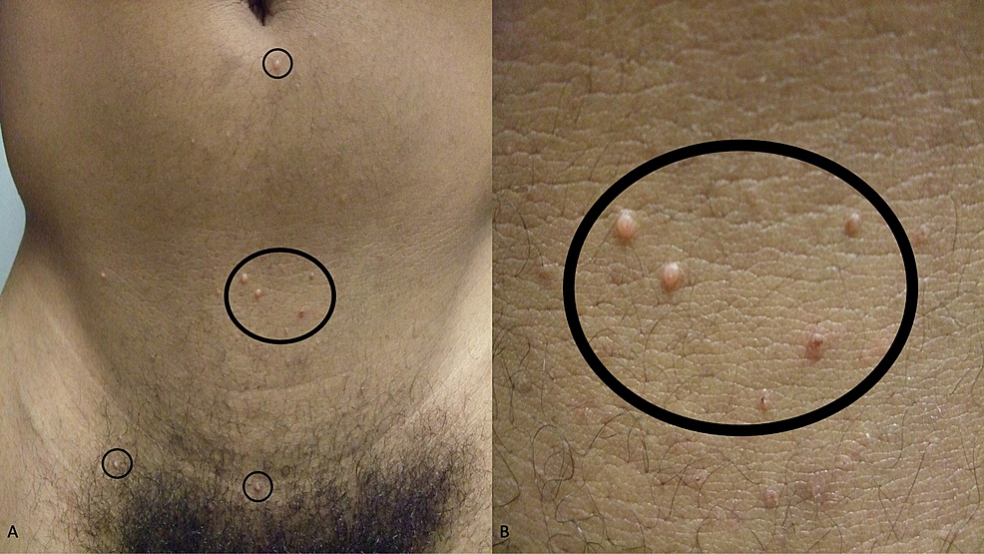
Molluscum contagiosum-associated white papules on the abdomen and suprapubic region Distal (A) and closer (B) views of individual and grouped lesions of molluscum contagiosum (within the black ovals) are present on the infraumbilical abdomen, the lower abdomen, and the hair-containing suprapubic region.

**Figure 3:**
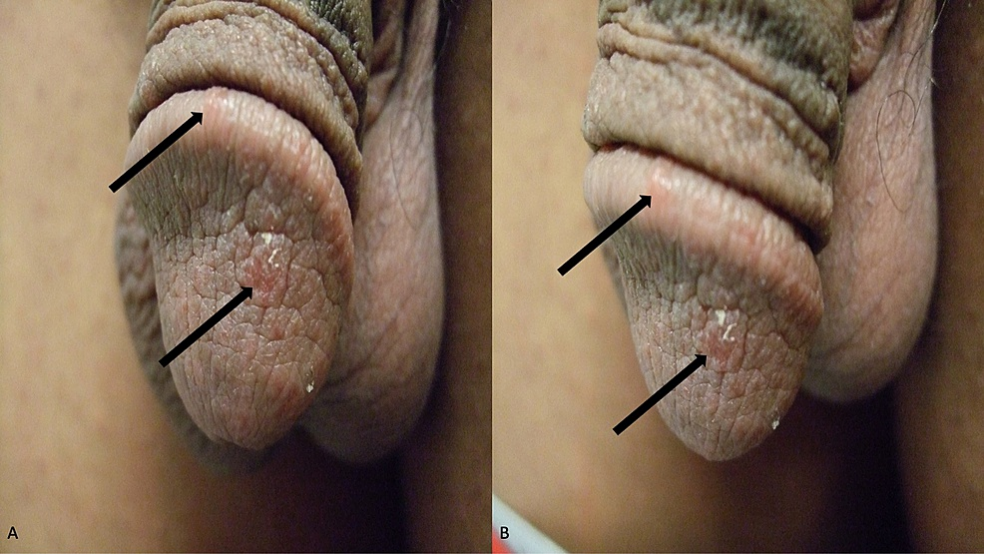
Sarcoptes scabiei var. hominis lesions on the penis Frontal (A) and left-side (B) views show erythematous scaly nodules of scabies on the glans and corona (black arrows) of a 20-year-old man’s penis.

Examination of his abdomen and groin showed multiple, individual and grouped, papules on his abdomen below the umbilicus, his suprapubic region, and on the shaft of his penis (Figure 1). The papules were three to five millimeters in diameter, white, and umbilicated (Figure 2). In addition, on small, five-millimeter, red scaling nodules were present on the glans and corona of his penis (Figure 3).

**Figure 4:**
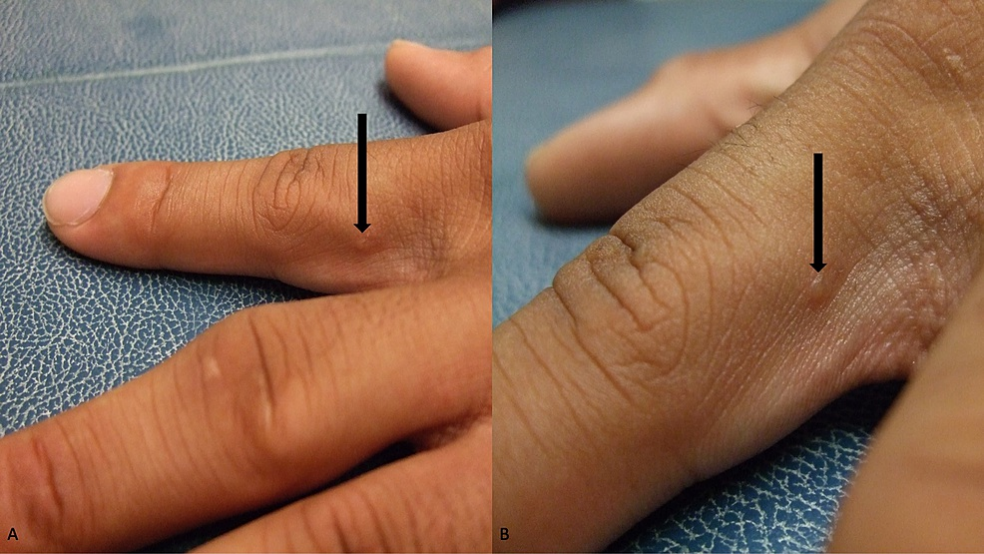
Scabetic burrow on the proximal left index finger Distal (A) and closer (B) views of a scabies-associated burrow (black arrow) on the ulnar side of the second digit on the left hand adjacent to the finger web. A scabies (mineral oil) preparation demonstrated not only scabies mite eggs but also scybala (scabies mite feces).

The umbilicated papules were suggestive of molluscum contagiosum; their location was consistent with being acquired sexually. The red penile nodules raised the possibility of scabies; therefore, a complete evaluation of his skin was performed. A burrow was identified on the lateral side of his left index finger just distal to the finger web between the second and third digit (Figure 4).

A molluscum preparation was performed by curetting one of the abdominal umbilicated papules and spreading its contents on a glass slide followed by the application of a stain containing toluidine blue and basic fuchsin in 30 percent alcohol. Molluscum bodies (also referred to as Henderson-Patterson bodies) were observed using a light microscope. This confirmed the diagnosis of molluscum contagiosum infection.

A scabies (mineral oil) preparation was performed by applying mineral oil to the burrow on his finger and scraping the lesion with a number 15 blade; the material obtained was gently smeared onto a glass microscope slide. Not only scabies mite eggs, but also scybala (mite feces) were observed using a light microscope. This confirmed the diagnosis of *Sarcoptes scabiei* infestation presenting with a typical burrow and penile nodules (scabies surrepticius).

Additional history was subsequently provided by the patient. About six weeks earlier, he had met a woman at a party. They had a single sexual encounter that evening; he did not use a condom. He had not seen the person since that night.

Laboratory testing to rule out other potential sexually transmitted diseases was performed. Studies included evaluation for chlamydia, gonorrhea, syphilis, hepatitis B, hepatitis C, and human immunodeficiency virus (HIV). All the studies were negative.

The molluscum contagiosum lesions were each treated by removal using curettage. His scabies infestation was treated by applying permethrin five percent cream before sleep from his neck to his toes and washing the medication off with water in a shower the following morning; the treatment was repeated in seven days. Neither lesions of molluscum contagiosum nor scabies were observed at a follow-up appointment four weeks after the initial visit.

## Discussion

Molluscum contagiosum is a commonly occurring viral infection of the skin. It is frequently observed in younger children and acquired non-sexually by incidental contact of a disease-free individual with the infected person. The lesions are often located on the arms and legs, the chest and abdomen, and occasionally the face. However, molluscum contagiosum also occurs in older individuals who often acquire the infection during sexual activity; in contrast to infected children, the older patients have lesions that are found on the genitalia, groin, suprapubic area, lower abdomen, and proximal thighs [1,3].

The diagnosis of molluscum contagiosum can be suspected based on the clinical morphology of the skin lesions. Although only a solitary lesion may be present, multiple (individual or grouped) lesions frequently occur. The papules typically vary in size from one to five millimeters, are white to flesh-colored, and are dome-shaped; a central umbilication is often present--at least in some of the papules [2,4].

Dermoscopy is a non-invasive method to assist in identifying the suspected papule as molluscum contagiosum; however, the technique is dependent on the experience of the clinician and the availability of a dermatoscope. Centrally, in the umbilicated area, polylobular, roundish or four-leaved clover-like, white and/or yellow, amorphous structures (referred to as clods and representing molluscum bodies) can be observed. Crown vessels (which are red, linear curved vessels with minimal branching) can be noted at the periphery [1,8,9].

A molluscum preparation, the same as that which was used to confirm the diagnosis for the man in this report, is a rapid and simple procedure that can readily be performed by the clinician. The central portion of the papule (obtained after curettage of the lesion or expressed after superficially cutting the papule’s surface with a scalpel blade) is crushed onto a microscope slide and stained with either giemsa, gram, hematoxylin and eosin, methylene blue, Papanicoulaou, toluidine blue or Wright stain for 30 to 60 seconds. After the excess stain is gently washed off with water, a cover slip is applied and examination using a light microscope shows numerous, darkly staining, amorphous molluscum bodies [10].

A biopsy of one or more lesions--obtained using either curettage, punch, or shave technique--can also be performed to establish the diagnosis. The specimen is placed in formalin, processed, and usually stained with hematoxylin and eosin. Distinctive and pathognomonic changes consisting of large eosinophilic round molluscum bodies in the basal layers of the epidermis, which appear basophilic in the epidermal upper layers, confirm the diagnosis of molluscum contagiosum [10].

Treatment options for molluscum contagiosum include observation since spontaneous resolution may occur. However, many patients--and especially older sexually active individuals--prefer interventional therapy. These modalities include mechanical methods, chemical methods, immunomodulatory methods, and antiviral therapy [1,2,4,11,12].

The molluscum contagiosum lesions of the patient in this report were treated using a mechanical technique: curettage. All his lesions were removed at a single treatment session and additional lesions had not developed when he returned for follow-up examination one month later. Other mechanical methods for treating molluscum contagiosum include cryotherapy with liquid nitrogen and lesion ablation using (pulse dye) laser therapy [1,4,11].

Chemical methods of molluscum contagiosum management consist of the application of a topical agent. An inflammatory response occurs at the site or treatment with subsequent eradication of the viral lesion. Some of the agents that have been used include benzoyl peroxide, cantharidin, glycolic acid, lactic acid, podophyllotoxin, potassium hydroxide, salicylic acid, tretinoin, and trichloroacetic acid [1,2,4].

Immunomodulatory methods to treat molluscum contagiosum result in therapy-associated lymphocyte recruitment and cytokine release. This results in destruction and/or regression of the viral lesions. These include cimetidine (an orally administered histamine type two receptor antagonist), diphencyprone (a topical agent that causes a marked sensitization reaction), and interferon-alpha (intralesionally or subcutaneously). Other intralesional therapies for molluscum contagiosum have also been used: Candida antigen, combined mumps, measles, rubella (MMR) vaccine, Streptococcal substrain OK-432, tuberculin purified protein derivative (PPD), and vitamin D3. Topical imiquimod was also previously used; however, several investigators no longer advocate its use to treat molluscum contagiosum [1,2,12].

Antiviral treatment for molluscum contagiosum has included cidofovir. Cidofovir--topically or intravenously--has been reported to be effective in some immunosuppressed patients with refractory lesions. Adverse effects to the drug include nephrotoxicity when received intravenously; topical therapy can be associated with erosions, irritation, pigmentary changes, and scars at the application sites [1,2,4].

Burrows, linear and raised scaly lesions, are the classical skin lesions of scabies. Similar to the patient in this report, burrows most commonly occur on the arms (and particularly the finger webs, hands, and wrists) and legs (including the feet). Other common sites include the axillae, breasts, and buttocks. Primary scabetic lesions can also present as small scaly papules like those noted on the penis of the man in this report. They can also present with nodules on the scrotum or other genital areas [5-7].

In addition, pruritus--not only of the lesions but also generalized and thereby involving the entire body--typically develops. The itching is often worse at night. In addition, it is common for the patient’s close personal contacts, such as family members, to have already become infected with scabies and therefore also itch [6,7].

There are various non-classic presentations of scabies. Some of these variants can mimic other conditions such as bullous pemphigoid, dermatitis, and prurigo nodularis; indeed, the lesions can be blisters, crusted plaques, and nodules. Indeed, in some individuals, the lesions are located on the scalp. Recently, these non-classic variants of scabies have collectively been referred to as scabies surrepticius [5].

Genital scabies can occur as an isolated manifestation of a scabies infestation; however, similar to the man in this report, scabies lesions on the genitalia more commonly accompany other mite-associated lesions at other locations. Not only burrows but also papules or nodules may be present on the glans penis, penile shaft and/or scrotum. The nodular lesions may contain mites, or they can be parasite-free and represent a hypersensitivity reaction to the antigens of the mite [13,14].

Non-invasive and invasive methods are available for diagnosing scabies. Applying ink or a marker pen to a suspected lesion and removing the excess agent with alcohol can be used to identify the end of a burrow. Alternatively, a suspected lesion can be removed with adhesive tape and then the skin-containing tape is placed on a microscope slide; a light microscope is used to examine the slide for mites, mite eggs, or scybala. These are simple, yet often unsuccessful, methods to confirm a scabies diagnosis. Similarly, sophisticated equipment to visualize burrows and mites--such as videodermoscopy, reflectance confocal microscopy, and optical coherence tomography--are not routinely available for diagnostic use in most clinical settings [6].

Dermoscopy can be used to assess for the presence of scabies mites. It is a non-invasive technique with an essentially equivalent sensitivity (91%) as compared to skin scraping (90%) for diagnosing scabies. However, similar to its use in evaluating patients for molluscum contagiosum, the technique requires the presence of a dermatoscope and is highly dependent on the clinical expertise of the individual doing the evaluation [8,9].

Several terms have been used to describe the dermoscopic appearance of the black, triangular-appearing, head of the mite: triangle or delta-wing jet or delta glider or hang glider sign or spermatozoid sign. The mite’s abdomen and eggs are translucent and therefore not visualized with dermoscopy. The conical sign refers to the visualization of the burrow as a thin track or white scale that is noted extending from the mite’s head [5,8,9].

Skin scraping, to perform a scabies (mineral oil) preparation, is the gold standard for diagnosing scabies. Similar to the patient in this report, mineral oil is placed on the suspected lesion and the site is scraped with a number 15 blade to remove the superficial layers of the epidermis. The specimen is gently smeared onto a microscope slide containing mineral oil. A cover slip is applied, and a light microscope is used to examine the slide. The presence of a mite, an egg, and/or scybala confirms the suspected diagnosis of a scabies infestation [5-7].

A skin biopsy--either using the shave or punch technique--can also be used to establish the diagnosis of scabies. This method of diagnosis often occurs in patients for whom a scabies infestation is not clinically suspected. The discovery of one or more mites in the stratum confirms the scabies diagnosis [5,6].

A rapid and confirmatory point-of-care laboratory-based test for establishing the diagnosis of scabies is currently being sought. Serology assays and molecular-based techniques (such as DNA isolation, conventional polymerase chain reaction, real-time polymerase chain reaction, and isothermal amplification techniques) have recently been developed that can be used for the diagnosis of scabies. However, to date, none of these methods have progressed from the realm of a research finding to being utilized in clinical practice [15].

In 2018, the International Alliance for the Control of Scabies (IACS) proposed criteria for the diagnosis of scabies. Although the criteria were originally established for scabies research, they have been adopted for use by clinicians evaluating patients. The diagnosis of scabies can either be confirmed, clinical, or suspected [5,6].

A confirmed scabies diagnosis, similar to the patient in this report, is provided by observing either the mite, eggs, or scybala on light microscopy (of a mineral oil preparation or skin biopsy) or a high-powered imaging device; alternatively, the diagnosis is confirmed by visualizing the mite on dermoscopy. A clinical scabies diagnosis requires either the observation of burrows or genital lesions (in men) or typical lesions (in morphology and location) along with two historic scabies features (which include both pruritus and close contact with a person who either itches or has typical appearing and distributed scabies lesions). And, a suspected scabies diagnosis may be made if there are either typical lesions (in morphology and location) and one historic feature or lesions that are atypical (in morphology or location) and two historic features [5,6].

The agents available for the treatment of scabies include topical medications and a systemic drug. The advantages of topical agents are that some can be used in young children, pregnant women, and nursing mothers; disadvantages of these agents include not only that some require repeat treatments, but also the challenges a patient may experience in applying them to their bodies--especially to sites that are difficult to reach. The advantages of oral ivermectin therapy are the ease with which it can be used by the patient; however, disadvantages are the specific patient populations who should not use the drug and that the treatment needs to be repeated to ensure that all of the mites have been eliminated since the medication is not ovicidal [5-7,16].

The most commonly used topical therapy for scabies, which kills both mites and eggs, is five percent permethrin. It is approved for use in infants older than two months. It has also been used during pregnancy but is not recommended for women who are breastfeeding [5-7,16].

Permethrin cream is applied from the neck to the toes overnight (for at least eight hours) and washed off the following morning. It is important to treat all areas of the body including the umbilicus, the skin surfaces of the genitals, the cutaneous perianal area, and beneath all fingernails and toenails. In people with alopecia of their head hair, it may be prudent to also treat the scalp [5-7,16].

The permethrin treatment should be repeated in one week. The scabies infestation of the patient in this report was successfully treated with two applications (one week apart) of permethrin five percent cream [5-7,16].

Other topical therapies for the treatment of scabies include benzyl benzoate, crotamiton, and precipitated sulfur; these agents are safe in children older than two months and for women during pregnancy. Malathion is another topical agent. In addition, topical ivermectin (0.5 percent lotion for head lice and one percent topical cream for rosacea) has also been used. Gamma benzene hexachloride one percent lotion (lindane) was a prior topical agent used to treat scabies; however, it was found to be carcinogenic and its use has been banned by several countries [5-7,16].

Spinosad 0.9 percent lotion has recently been approved for treating scabies in patients aged four years and older. It is an insecticide derived from the soil acinobacterium Saccharopolyspora spinosa. Its insecticidal mechanism of action involves functional alteration of the nicotinic and gamma-aminobutyric acid-gated ion channels causing nervous system excitation in insects; this is favored to be how the agent kills scabies mites [17].

Spinosad has previously been successfully used to treat head lice. Only a single treatment is necessary to treat scabies; the lotion is applied from neck to toes (and on the scalp if the person is bald) for at least six hours before washing it off. Evaluation 28 days after the single treatment demonstrated a complete cure in 78.1 percent of the patients [17].

Ivermectin is an oral agent used in the treatment of scabies. The drug is also used in the management of other helminth parasites that cause onchocerciasis and strongyloidiasis. Ivermectin is not recommended for pregnant women, nursing mothers, children less than five years old, and infants less than 15 kilograms [5-7,16].

The dose of ivermectin is 200 micrograms per kilogram (which is the same as 0.2 milligrams per kilogram). Since the drug is not ovicidal, treatment needs to be repeated in seven days. However, for patients with crusted scabies (in whom thousands of mites may be present), multiple doses--either three, five, or seven--administered on two consecutive days each week for up to four weeks may need to be given [5-7,16].

Persistent scabies infestation can occur secondary to emerging resistance to topical therapies. However, it is more likely to be the sequalae of inadequate application of a topical agent to all body sites--especially in areas that cannot be reached such as the middle of the back, or the scalp, or under the nails. In addition, what is interpreted as a persistent mite infestation may indeed be a new infestation of scabies acquired from a close contact, such as a family member, who was not concurrently treated when the patient received therapy and thereby was the source of the perceived unresponsive scabies infection of the patient [5,6].

Sexually transmitted diseases describe contagious conditions that can be acquired by a disease-free individual during a sexual encounter with an infected partner. However, some of these conditions can also be transmitted in a non-sexual interaction between the infected individual and the unsuspecting recipient. Many of the diseases have clinical features that affect the genitalia, yet others result in systemic manifestations [18-20].

Similar to the man in this report, an intimate conjugal relationship was a common risk factor for the acquisition of molluscum contagiosum and scabies. The classic morphologic features of molluscum contagiosum (white to flesh-colored umbilicated papules) and scabies (burrows and genital nodules) are distinctive. Indeed, there were no common clinical or dermoscopic features of molluscum contagiosum infection and scabies infestation [1-9].

There are several conditions that are classified as sexually transmitted diseases. Reportable sexually transmitted infections to the US Center for Disease Control (CDC) national notifiable diseases surveillance system include chancroid, chlamydia, gonorrhea, hepatitis A, hepatitis B, hepatitis C, HIV, syphilis, and Zika virus [18]. Other sexually transmitted diseases include bacterial vaginosis, herpes simplex virus infection, human papillomavirus infection, lymphogranuloma venereum, molluscum contagiosum, non-gonococcal urethritis (*Mycoplasma genitalium* infection), pediculosis, scabies, and trichomonas vaginalis [19,20].

The man in this report presented with two conditions (molluscum contagiosum which has an incubation period of two to six weeks and scabies which has an incubation period of four to eight weeks) that he acquired during a single sexual encounter six weeks earlier: two for one. He was evaluated for other potential sexually transmitted diseases including chlamydia, gonorrhea, hepatitis B, hepatitis C, HIV, and syphilis. He had a complete skin examination and there were no lesions of herpes simplex virus infection (such as grouped vesicles on an erythematous base) or human papillomavirus infection (such as condyloma acuminatum) [1,6].

Some of the sexually transmitted diseases are recommended to be screened for in certain patient groups. Bacterial vaginosis may be considered for screening in asymptomatic pregnant women at high risk for preterm delivery. In men who have sex with men, the CDC recommends vaccination for hepatitis A, hepatitis B, and human papillomavirus and annual screening for anogenital warts (anal cytology) and anal squamous cell neoplasms, chlamydia (rectal, and urine nucleic acid amplification test), gonorrhea (pharyngeal, rectal, and urine nucleic acid amplification test), HIV, and syphilis. Screening for women who have sex with women and transgender persons should be guided by their sexual history [18].

## Conclusions

Molluscum contagiosum is a viral infection that presents with white to flesh-colored, often umbilicated, papules; in sexually active adolescent and older individuals it can be acquired during intercourse and the lesions develop on the groin and genitalia. Human scabies is a mite infestation caused by Sarcoptes scabiei var. hominis. The scabies infestation is acquired by contact with an infected individual; similar to molluscum contagiosum, scabies can also be transmitted during a sexual interaction. A disease-free 20-year-old man concurrently developed molluscum contagiosum infection and scabies infestation following a single sexual encounter with an infected female partner. The diagnosis of molluscum contagiosum was established by observing molluscum bodies on the molluscum preparation from the abdominal papules. The diagnosis of scabies was established by observing mite eggs along with scybala on the scabies preparation taken from the index finger burrow. The viral infection and mite infestation were both successfully treated; the molluscum contagiosum lesions were curetted and the scabies infestation was treated with two topical treatments (each one week apart) using five percent permethrin cream. The discovery of a single sexually transmitted disease should prompt the clinician to evaluate the patient for the potential concurrent acquisition of additional conditions that can be transmitted sexually.
